# The Association Between Previous TORCH Infections and Pregnancy and Neonatal Outcomes in IVF/ICSI-ET: A Retrospective Cohort Study

**DOI:** 10.3389/fendo.2020.00466

**Published:** 2020-08-05

**Authors:** Yifeng Liu, Yiqing Wu, Feixia Wang, Siwen Wang, Wei Zhao, Lifen Chen, Shijiong Tu, Yuli Qian, Yun Liao, Yun Huang, Runjv Zhang, Gufeng Xu, Dan Zhang

**Affiliations:** ^1^Key Laboratory of Reproductive Genetics (Ministry of Education), Department of Reproductive Endocrinology, School of Medicine, Women's Hospital, Zhejiang University, Hangzhou, China; ^2^Women's Reproductive Health Research Key Laboratory of Zhejiang Province, Hangzhou, China; ^3^Department of Obstetrics, Gynecology, and Reproductive Sciences, Yale School of Medicine, New Haven, CT, United States; ^4^Department of Obstetrics, Gynecology and Reproductive Biology, Brigham and Women's Hospital, Harvard Medical School, Boston, MA, United States

**Keywords:** TORCH, previous infection, pregnancy outcome, neonatal outcome, IVF/ICSI-ET

## Abstract

**Objective:** This study aimed to investigate the associations between previous TORCH infection (cytomegalovirus, toxoplasmosis, herpes simplex virus, and rubella) with pregnancy and neonatal outcomes in couples undergoing IVF/ICSI-ET.

**Materials and Methods:** A total of 18,074 couples underwent fresh IVF/ICSI-ET (*in vitro* fertilization/intracytoplasmic sperm injection–embryo transfer) cycles were included in our analyses. TORCH infection status was determined by serological confirmation of cytomegalovirus, toxoplasmosis, herpes simplex virus, and rubella IgG in the absence of IgM antibodies. Clinical pregnancy, ectopic pregnancy, miscarriage, live birth, preterm birth, congenital malformation, and perinatal death were evaluated in both infection and non-infection group. Multivariate logistic regression was applied to calculate odds ratio.

**Results:** Previous toxoplasmosis infection is associated with a significantly decreased preterm birth rate [*P* = 0.045, OR = 0.755 (95% CI, 0.571–0.997), Adjusted OR = 0.749 (95%CI, 0.566–0.991)]. No differences in clinical pregnancy, ectopic pregnancy, miscarriage, and perinatal death were observed between the corresponding TORCH infection group [IgM (–) IgG(+)] and the non-infection group [IgM (–) IgG (–)].

**Conclusions:** Previous TORCH infections were not associated with adverse pregnancy and neonatal outcomes in IVF/ICSI-ET overall, and toxoplasmosis infection might be associated with a lower preterm birth rate in patients underwent IVF/ICSI-ET. The necessity of TORCH IgG screening in IVF procedure might need re-evaluation, and further cost-effective analysis might be helpful for the clinical management strategy.

## Introduction

TORCH infections classically comprise cytomegalovirus (CMV), toxoplasmosis, rubella, herpes simplex virus (HSV), and other commonly seen intrauterine infections, such as varicella and enteroviruses. The diseases caused by these pathogens can be transmitted from mothers to offspring (1) and are the main contributors to prenatal and neonatal abnormality and mortality (2).

CMV is a common viral pathogen which represents significant health concerns, as it could remain dormant in the host body over a long time, and may transmit from a mother to her developing fetus (3). It has been documented that congenital CMV infection affect ~0.3–2.0% of all newborns (4). The relationship between the CMV viral load and pregnancy and live-birth outcomes were suspected, with several previous studies showing that there was a positive correlation between high viral load and adverse clinical outcomes of the fetus (5, 6). *Toxoplasma gondii* infection during pregnancy may also result in severe fetal damage, which manifests as the classic triad of chorioretinitis, hydrocephalus, and intracranial calcifications with parasites transmitting through the placenta (7). Rubella virus infection during pregnancy predisposes the fetus to developing a constellation of congenital deformities known as congenital rubella syndrome (CRS) secondary to maternal infection, especially during the first trimester (8). Congenital HSV infection shares clinical features with other congenital infections, such as microcephaly, hydrocephalus, and chorioretinitis, and usually presents with clinical symptoms at birth, mainly due to exposure to HSV during delivery (9). Other pathogens like spirochete *Treponema pallidum* will cause Syphilis syndrome (10). Most of the pathogens mentioned above have common clinical features of rash and ocular abnormalities that bring a huge burden on healthcare system and the society (11).

The life cycle of TORCH agents are different from each other, and the infections of TORCH are believed to have lifelong influences. For CMV infection, lifelong latency is established after acute infection in infected hosts (12). The natural cycle of initial infection is related to an increased IgG level and decreased IgM level, while women with IgG-seropositive CMV infection could not be absolutely protected against reactivation or reinfection of the same pathogen (13). Besides, it has been reported that more children in the United States acquire congenital CMV infection from non-primary maternal infection than from primary maternal infection (14). Meanwhile, several studies have found that there is a link between human behavior, personality, or mental disorders and toxoplasmosis IgG seropositivity (15). A nested case-control study with age-matched participants found that fetal gastroschisis was associated with maternal HSV IgG reactivity (16). Rubella IgG is considered as a protective antibody from recurrent Rubella infection, so WHO and other guidelines strongly recommend individuals to take the Rubella vaccination reaching an IgG titer of 10 IU/mL anti-Rubella antibodies in serum (17). However, the influence of previous TORCH infection on long-term health, especially pregnancy and neonatal outcomes, is yet to be confirmed.

Since the infection rate of TORCH was relatively high among women at child-bearing age in Asia (18, 19), women are routinely checked for TORCH infection status, namely, IgM and IgG, before commencing IVF/ICSI cycles in our center. Nevertheless, there is no reliable information about the impact of previous TORCH infections on the outcomes of pregnancy and live birth in women undergoing IVF-ET. Hence, our study aims to investigate the association between past TORCH infections with IVF-ET outcomes.

## Materials and Methods

This retrospective, hospital-based cohort study was approved by the Hospital Ethics Committee, Women's Hospital, Zhejiang University School of Medicine. Since it is a retrospective chart review study with only de-identified information collected, the Ethics Committee of Women's Hospital, Zhejiang University School of Medicine had determined exemption for informed consent of the study participants.

**Figure 1:**
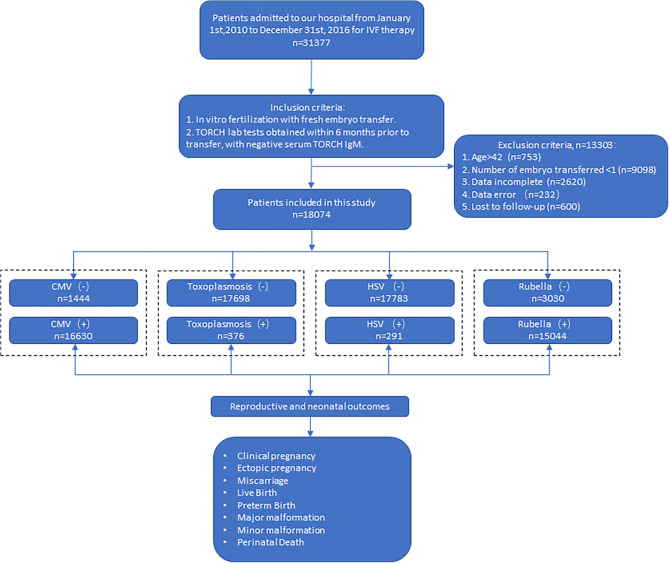
Flowchart of the study. CMV (–): CMV IgM (–) IgG (–); CMV (+): CMV IgM (–) IgG(+). Toxoplasmosis (–): toxoplasmosis IgM (–) IgG (–); roxoplasmosis (+): toxoplasmosis IgM (–) IgG (+). HSV (–): HSV IgM (–) IgG (–); HSV (+): HSV IgM (–) IgG (+). Rubella (–): rubella IgM (–) IgG (–); rubella (+): rubella IgM (–) IgG (+).

Patients admitted to our hospital for IVF treatment from January 1, 2010, to December 31, 2016, were enrolled. Inclusion criteria are as follows: (1) *in vitro* fertilization with fresh embryo transfer; (2) TORCH lab tests obtained within 6 months prior to transfer, with negative serum TORCH IgM. Exclusion criteria were (1) age more than 42 years old; (2) number of embryos transferred <1; (3) lost to follow-up; (4) incomplete data; and (5) data error. A total of 31,377 couples were recruited, and 18,074 were included in our final analysis. Flow chart was as in Figure 1.

Patients were divided into two groups, according to TORCH IgG seropositivity or not, clinical outcome data were collected and evaluated between the two groups. Primary outcomes were clinical pregnancy and live birth rate. Secondary outcomes were ectopic pregnancy, miscarriage, preterm birth, major malformation, minor malformation, and perinatal death. Clinical pregnancy was defined as the visualization of at least one gestational sac on ultrasound. Live birth was defined as baby born alive after 28 weeks of gestation. Ectopic pregnancy is defined as the development of a fertilized egg elsewhere than in the uterus. Miscarriage was defined as clinical pregnancy that was subsequently spontaneously miscarried. Preterm birth is defined as baby born alive before 37 weeks of gestation. Major malformations were defined as anomality that generally cause functional impairment or require surgical correction, and minor malformations were defined as the remaining malformations (20). Perinatal death was defined as infant death occur at less than 7 days of age or fetal death after 28 weeks of gestation. IVF and pregnancy outcomes were obtained from routine telephone follow-up by staff in our IVF center. Baseline characteristics were obtained from the running database in our IVF center. The rate of the primary and secondary outcomes were all calculated based on number of all cycles respectively.

Statistical analysis was performed using SPSS (Statistical Package for the Social Sciences Version 23.0, IBM Corp., Armonk, NY, USA). Chi-square test and Student's *t*-test were used to compare proportions and means respectively. Adjusted odds ratios (OR) with 95% confidence intervals (CI) were calculated to approximate relative risks of adverse outcomes. ORs were estimated using multivariate logistic regression for control of confounding factors. CMV infection, maternal age, paternal age, baseline FSH, number of MII oocytes, number of oocytes, number of 2PN created, and number of embryos transferred were factors put into the logistic regression model for previous CMV infection effect analysis. Toxoplasmosis infection, maternal age, paternal age, number of embryos transferred were the factors put into the logistic regression model for previous Toxoplasmosis infection effect analysis, while HSV infection, maternal age, paternal age, duration of infertility, number of embryos transferred were the factors used in the multivariate logistic regression for previous HSV infection effect analysis. As for the previous rubella infection effect analysis, rubella infection, maternal age, paternal age, antral follicle count, endometrial thickness, number of MII oocytes, and number of embryos transferred were used as factors for multivariate logistic regression model. The method of backward testing was used for the selection of independent variables in logistic regressions with entry *P* value = 0.05 and removal *P* value = 0.1. *P* < 0.05 (two-tailed) was considered statistically significant.

## Results

### Baseline Characteristics

Baseline characteristics of the study couples were shown in Table 1. There were differences in maternal age, paternal age, etiology of infertility, baseline FSH, number of MII oocyte, oocytes collected, 2PN created, and embryos transferred between the CMV IgM (–) IgG(+) group and CMV IgM (–) IIgG (–) group. All the baseline characteristics were similar between the toxoplasmosis IgM (–) IgG(+) group and toxoplasmosis IIgM (–) IgG (–) group. Baseline characteristics were similar between the HSV IgM (–) IgG(+) group and HSV IgM (–) IgG (–) group, except for duration of infertility, maternal BMI, and antral follicle count. As for the rubella IgM (–) IgG(+) group and rubella IgM (–) IgG (–) group, differences were found in maternal age, paternal age, previous pregnancy, etiology of infertility, endometrial thickness, number of MII oocytes, and whether at least one good embryo was transferred. All the factors with significant differences were put into the multivariate logistic regression analysis to adjust ORs.

**Table 1:** Baseline characteristics of couples.

	**CMV (–)**	**CMV (+)**	***P***	**Toxoplasmosis (–)**	**Toxoplasmosis** **(+)**	***P***	**HSV (–)**	**HSV (+)**	***P***	**Rubella (–)**	**Rubella (+)**	***P***
No of IVF cycles	1,444	16,630		17,698	376		17,783	291		3,030	15,044	
Female Age	31.73 ± 4.42	31.22 ± 4.41	0.000	31.26 ± 4.41	31.36 ± 4.54	0.684	31.26 ± 4.41	31.38 ± 4.25	0.659	31.66 ± 4.54	31.19 ± 4.38	0.000
Duration of infertility (Years)	4.48 ± 3.29	4.30 ± 3.15	0.056	4.31 ± 3.16	4.46 ± 3.20	0.385	4.32 ± 3.16	3.98 ± 2.75	0.037	4.36 ± 3.18	4.31 ± 3.15	0.412
Female BMI	22.10 ± 2.75	22.18 ± 2.88	0.347	22.17 ± 2.88	22.20 ± 2.81	0.810	22.18 ± 2.87	21.77 ± 2.82	0.017	22.24 ± 2.81	22.15 ± 2.89	0.120
Male Age	33.99 ± 5.48	33.38 ± 5.28	0.000	33.41 ± 5.30	33.96 ± 5.61	0.049	33.42 ± 5.30	33.58 ± 5.51	0.621	33.88 ± 5.41	33.33 ± 5.28	0.000
Male BMI	23.69 ± 3.03	23.74 ± 3.20	0.543	23.73 ± 3.17	23.92 ± 3.59	0.304	23.74 ± 3.19	23.67 ± 3.03	0.746	23.81 ± 3.18	23.72 ± 3.18	0.160
Previous pregnancy in couple			0.495			0.284			0.524			0.001
Yes No	818 (56.6) 626 (43.4)	9,266 (55.7) 7,364 (44.3)		9,864 (55.7) 7,834 (44.3)	220 (58.5) 156 (41.5)		9,927 (55.8) 7,856 (44.2)	157 (54.0) 134 (46.0)		1,772 (58.5) 1,258 (41.5)	8,312 (55.3) 6,732 (44.7)	
Main reason of infertility			0.008			0.969			0.878			0.000
Tubal Anovulatory Male factor Endometriosis Unexplained Others	745 (51.6) 50 (3.5) 296 (20.5) 154 (10.0) 79(5.5) 130 (9.0)	9,377 (56.4) 558 (3.4) 3,257 (19.6) 1,462 (8.8) 772 (4.6) 1,204(7.2)		9,905 (56.0) 598(3.4) 3,482 (19.7) 1,573 (8.9) 833(4.7) 1,307(7.4)	217 (57.7) 10(2.7) 71 (18.9) 33 (8.8) 18(4.8) 27(7.2)		9,962 (56.0) 597(3.4) 3,601 (19.7) 1,578 (8.9) 834 (4.7) 1,311(7.4)	160 (55.0) 11(3.8) 52 (17.9) 28 (9.6) 1 7(5.8) 23(7.9)		1,651 (54.5) 89(2.9) 593 (19.6) 336 (11.1) 130 (4.3) 231(7.6)	8,471 (56.3) 519(3.4) 2,960 (19.7) 1270 (8.4) 721 (4.8) 1,103(7.3)	
Baseline FSH	6.88 ± 2.64	7.04 ± 2.45	0.020	7.02 ± 2.46	7.02 ± 2.61	0.990	7.03 ± 2.47	6.78 ± 2.28	0.083	6.99 ± 2.39	7.03 ± 2.48	0.416
AFC	10.72 ± 4.62	10.94 ± 4.61	0.104	10.92 ± 4.62	10.88 ± 4.32	0.843	10.91 ± 4.61	11.45 ± 4.75	0.055	10.74 ± 4.63	10.96 ± 4.61	0.023
Em Thickness(mm)	10.85 ± 2.97	10.80 ± 2.39	0.483	10.81 ± 2.45	10.79 ± 2.28	0.900	10.81 ± 2.44	10.79 ± 2.38	0.915	10.73 ± 2.34	10.82 ± 2.46	0.045
No of MII oocyte	1.07 ± 3.21	1.71 ± 3.88	0.000	1.66 ± 3.84	1.46 ± 3.77	0.324	1.65 ± 3.83	1.75 ± 4.13	0.664	1.51 ± 3.65	1.68 ± 3.87	0.024
Type of fertilization			0.528			0.588			0.338			0.358
IVF ICSI	1,021 (70.7) 423 (29.3)	11,775 (70.8) 4,855 (29.2)		12,536 (70.8) 5162 (29.2)	260 (69.1) 116 (30.9)		12,585 (70.8) 5,198 (29.2)	211 (72.5) 80 (27.5)		2,115(69.8)916(30.2)	10,682 (71.0) 4,362 (29.0)	
No of eggs collected	11.30 ± 5.88	10.91 ± 5.62	0.010	10.94 ± 5.64	10.68 ± 5.85	0.368	10.94 ± 5.65	10.71 ± 5.30	0.491	10.85 ± 5.66	10.96 ± 5.64	0.325
No of2PN created	6.78 ± 4.44	6.32 ± 4.10	0.000	6.36 ± 4.13	6.26 ± 4.28	0.621	6.36 ± 4.15	6.17 ± 3.85	0.434	6.34 ± 4.25	6.36 ± 4.11	0.774
No of embryo transfer	2.04 ± 0.61	1.94 ± 0.58	0.000	1.95 ±−0.59	1.96 ± 0.64	0.742	1.95 ± 0.59	1.97 ± 0.58	0.548	1.97 ± 0.60	1.95 ± 0.58	0.037
At least one good embryo transferred	815 (56.4)	9,433 (56.7)	0.835	10,047 (56.8)	201 (53.5)	0.200	10,074 (56.6)	174 (59.8)	0.283	1,677 (55.3)	8,571 (57.0)	0.099

*Values are Mean ± Standard Deviation or N(%)*.

*CMV (–):CMV IgM (–) IgG (–); CMV(+): CMV IgM (–) IgG(+)*.

*Toxoplasmosis(–):toxoplasmosis IgM (–) IgG (–); toxoplasmosis (+): toxoplasmosis IgM (–) IgG(+)*.

*HSV(–):HSV IgM (–) IgG (–); HSV (+): HSV IgM (–) IgG(+)*.

*Rubella(-):rubella IgM (–) IgG (–); rubella (+): rubella IgM (–) IgG(+)*.

### Reproductive and Neonatal Outcome in the Presence of CMV IgG

We investigated the differences in reproductive and neonatal outcomes between the CMV IgM (–) IgG(+) group and CMV IgM (–) IgG (–) group. As shown in Table 2, CMV IgM (–) IgG(+) group showed a lower live birth rate compared with CMV IgM (–) IgG (–) group (31.8 vs. 34.2%), while no statistically significant difference was noticed [*P* = 0.063, OR = 0.948 (95% CI, 0.895–1.003), adjusted OR = 0.965 (95%CI, 0.911–1.023)]. There was no significant difference regarding the rate of clinical pregnancy, ectopic pregnancy, miscarriage, preterm birth, major malformation, minor malformation, and perinatal death between the two groups.

**Table 2:** Crude and adjusted ORs for reproductive and neonatal outcome by presence of IgG of CMV.

	**CMV (–) N (%)**	**CMV (+) N (%)**	***P***	**Crude ORs (95% CI)**	**Adjusted ORs (95% CI)**
Clinical pregnancy	612 (42.4)	7,009 (42.1)	0.862	0.995 (0.942–1.051)	1.016 (0.961–1.074)
Ectopic pregnancy	26 (1.8)	383 (2.3)	0.218	1.134 (0.928–1.386)	1.167 (0.954–1.427)
Miscarriage	80 (5.5)	997 (6.0)	0.484	1.043 (0.927–1.172)	1.070 (0.951–1.204)
Live Birth	494 (34.2)	5,294 (31.8)	0.063	0.948 (0.895–1.003)	0.965 (0.911–1.023)
Preterm Birth	90 (6.2)	970 (5.8)	0.535	0.965 (0.863–1.079)	0.996 (0.890–1.115)
Major malformation	6 (0.4)	69 (0.4)	0.997	0.999 (0.658–1.518)	1.011 (0.664–1.538)
Minor malformation	1 (0.1)	25 (0.2)	0.719	1.474 (0.542–4.006)	1.517 (0.557–4.130)
Perinatal Death	3 (0.2)	33 (0.2)	0.763	0.977 (0.541–1.766)	1.038 (0.574–1.879)

*Clinical pregnancy, ectopic pregnancy, miscarriage, live birth, preterm birth, major malformation, minor malformation, and perinatal death were calculated based on number of all cycles*.

*CMV (–):CMV IgM (–) IgG (–); CMV(+): CMV IgM (–)IgG(+)*.

### Reproductive and Neonatal Outcome in the Presence of Toxoplasmosis IgG

The comparison of the differences in reproductive and neonatal outcomes between the toxoplasmosis IgM (–) IgG(+) group and toxoplasmosis IgM (–) IgG (–) group were shown in Table 3. Previous toxoplasmosis infection significantly lowered the preterm birth rate [*P* = 0.045, OR = 0.755 (95% CI, 0.571–0.997), Adjusted OR = 0.749 [95%CI, 0.566–0.991)], while no significant differences were identified in clinical pregnancy, ectopic pregnancy, miscarriage, live birth, major malformation, neonatal minor malformation, and perinatal death between the two groups.

**Table 3:** Crude and adjusted ORs for reproductive and neonatal outcome by presence of IgG of Toxoplasmosis.

	**Toxoplasmosis (–) N(%)**	**Toxoplasmosis (+) N(%)**	***P***	**Crude ORs (95% CI)**	**Adjusted ORs (95% CI)**
Clinical pregnancy	7,474 (42.2)	157 (39.1)	0.223	0.937 (0.844–1.040)	0.937 (0.843–1.042)
Ectopic pregnancy	402 (2.3)	7 (1.9)	0.597	0.903 (0.620–1.317)	0.899 (0.617–1.312)
Miscarriage	1,059 (6.0)	18 (4.8)	0.332	0.889 (0.700–1.128)	0.884 (0.696–1.122)
Live Birth	5,676 (32.1)	112 (29.8)	0.348	0.948 (0.848–1.060)	0.948 (0.847–1.062)
Preterm Birth	1,047 (5.9)	13 (3.5)	0.045	0.755 (0.571–0.997)	0.749 (0.566–0.991)
Major malformation	75 (0.4)	0 (0)	0.411	–	–
Minor malformation	24 (0.1)	2 (0.5)	0.101	1.984 (0.963–4.089)	1.980 (0.960–4.083)
Perinatal Death	36 (0.2)	0 (0)	1.000	-	-

*Ectopic pregnancy, miscarriage, live birth, preterm birth, major malformation, minor malformation, and perinatal death were calculated based on number of all cycles*.

*Toxoplasmosis (–):toxoplasmosis IgM (–) IgG (–); toxoplasmosis (+): toxoplasmosis IgM (–)IgG(+)*.

### Reproductive and Neonatal Outcome in the Presence of HSV or Rubella IgG

Comparison between previous HSV or Rubella infection and non-infection group were shown in Tables 4, 5, respectively. No differences in clinical pregnancy, ectopic pregnancy, miscarriage, live birth, preterm birth, offspring malformation, and perinatal death were noticed.

**Table 4:** Crude and adjusted ORs for reproductive and neonatal outcome by presence of IgG of HSV.

	**HSV (–) N(%)**	**HSV (+) N(%)**	***P***	**Crude ORs (95% CI)**	**Adjusted ORs (95% CI)**
Clinical pregnancy	7,495 (42.1)	126 (43.3)	0.693	1.024 (0.911–1.151)	1.019 (0.905–1.157)
Ectopic pregnancy	399 (2.2)	10 (3.4)	0.175	1.245 (0.905–1.713)	1.241 (0.902–1.709)
Miscarriage	1063 (6.0)	15 (4.8)	0.404	0.892 (0.680–1.168)	0.891 (0.679–1.167)
Live Birth	5,690 (32.0)	98 (33.7)	0.542	1.039 (0.919–1.174)	1.033 (0.912–1.170)
Preterm Birth	1,041 (5.9)	19 (6.5)	0.627	1.060 (0.838–1.340)	1.050 (0.829–1.329)
Major malformation	74 (0.4)	1 (0.3)	1.000	0.908 (0.338–2.441)	0.902 (0.336–2.424)
Minor malformation	26 (0.1)	0 (0)	1.000	–	–
Perinatal Death	36 (0.2)	0 (0)	1.000	–	–

*Ectopic pregnancy, miscarriage, live birth, preterm birth, major malformation, minor malformation, and perinatal death were calculated based on number of all cycles*.

*HSV (–):HSV IgM (–) IgG (–); HSV (+): HSV IgM (–) IgG(+)*.

**Table 5:** Crude and adjusted ORs for reproductive and neonatal outcome by presence of IgG of Rubella.

	**Rubella (–) N (%)**	**Rubella (+) N (%)**	***P***	**Crude ORs (95% CI)**	**Adjusted ORs (95% CI)**
Clinical pregnancy	1,281 (42.3)	6,340 (42.1)	0.891	0.997 (0.959–1.037)	0.982 (0.941–1.024)
Ectopic pregnancy	63 (2.1)	346 (2.3)	0.456	1.053 (0.919–1.206)	1.020 (0.886–1.174)
Miscarriage	188 (6.2)	889 (5.9)	0.531	0.974 (0.898–1.057)	0.975 (0.896–1.062)
Live Birth	962 (31.7)	4,826 (32.1)	0.722	1.008 (0.966–1.051)	0.992 (0.948–1.037)
Preterm Birth	175 (5.8)	885 (5.9)	0.819	1.010 (0.929–1.098)	1.003 (0.919–1.096)
Major malformation	15 (0.5)	61 (0.4)	0.659	0.937 (0.700–1.253)	0.931 (0.688–1.259)
Minor malformation	5 (0.2)	21 (0.1)	0.736	0.920 (0.564–1.498)	0.889 (0.544–1.453)
Perinatal Death	6 (0.2)	30 (0.2)	0.987	1.004 (0.647–1.556)	1.217 (0.722–2.053)

*Ectopic pregnancy, miscarriage, live birth, preterm birth, major malformation, minor malformation, and perinatal death were calculated based on number of all cycles*.

*Rubella (–):rubella IgM (–) IgG (–); rubella (+): rubella IgM (–) IgG(+)*.

## Discussion

Infection during pregnancy has long been proven to cause adverse pregnancy outcomes, such as abortion and disastrous sequelae depending on the pathogens. The most well-known group of teratogenic pathogens are referred to as “TORCH” (*Toxoplasma gondii*, others like *Treponema pallidum*, rubella virus, cytomegalovirus, herpes simplex virus), for which, up to now, the underlying mechanisms were still unclear (21). In our study, we found that there is no difference in clinical pregnancy, ectopic pregnancy, miscarriage, and perinatal death between the corresponding previous TORCH infection group and the non-infection group respectively. Previous toxoplasmosis infection is associated with a significantly decreased preterm birth rate.

Previous studies revealed that the crucial mechanisms of intrauterine infections leading to abortion, lowering live birth rate, and increasing risk of congenital malformations is possibly through upregulated oxidative stress and apoptosis pathways to inhibit placenta development and fetal growth (22–24). Patients admitted for IVF/ICSI-ET treatment will undergo a series of assisted reproductive technology to achieve pregnancy and are required to take a TORCH test before starting IVF/ICSI-ET cycles. With the number of infertile patients annually increasing, the cycles of IVF/ICSI-ET raised consequently, and ever since the administration of TORCH screening during or before pregnancy, more and more serologic positive patients have been identified (18). However, no preceding research has focused on investigating the effect of previous TORCH infection on patients undergoing IVF/ICSI-ET and the clinical significance of carrying out such screening in IVF centers. The lack of high-quality studies in this field places clinicians in a dilemma of patient management and decision making, which warrants more in-depth research.

It is reported that ~30% of primary CMV infections presented a positive IgM, but there was a high false-positive rate. Thus, the IgG test was preferred as a diagnostic test 3 to 4 weeks after initial exposure. The results of serologic testing become meaningful when there is seroconversion from IgG negative to positive or the IgG titer rises greater than 4-fold from baseline (25). Hence, we want to explore the correlation between IgG seropositivity and pregnant and neonatal outcomes among infertile patients receiving IVF/ICSI-ET treatment.

CMV is the most prevalent congenital viral infection worldwide, influencing up to 2.0% pregnancies (26). The total birth incidence of congenital CMV infection is estimated to be 0.64%, and the risk of primary CMV infection in seronegative mothers ranges between 0.7 and 4.1% (27). In our study, we found patients with previous CMV infection had a lower live birth rate in comparison with those without CMV infection after IVF/ICSI-ET [*P* = 0.063, OR = 0.948 (95% CI, 0.895–1.003), adjusted OR = 0.965 (95%CI, 0.911–1.032)], indicating that previous CMV infection might have an adverse effect on live birth rate in patients undergoing IVF/ICSI-ET. Although the difference was not significant, the trend would remind clinicians to pay more attention to previous CMV infection. What's more, from the results, we can interpret that there are no statistically significant differences in clinical pregnancy rate or miscarriage rate between the CMV IgG(+) group and CMV IgG (–) group. It has been reported that the mechanism of intrauterine CMV infection causing pregnancy loss is mainly through placental inflammation. Scientists had detected higher levels of multiple cytokines and growth factors in amniotic fluid from those who with CMV infection than uninfected controls (21). The results observed in our study could possibly be caused by a similar pathophysiological effect of CMV IgG antibodies. Therefore, prevention of CMV infection seems to be more important during preparation for pregnancy, which incorporates hygiene precautions and behavioral interventions based on published findings, such as thoroughly washing hands with soap and water for 15 to 20 s (28). Further studies are needed to explore the effect of different levels of IgG avidity on pregnant and neonatal outcomes among patients underwent IVF/ICSI-ET.

*Toxoplasma gondii* is a similarly prevalent infection throughout the world. *Toxoplasma gondii* is a protozoan parasite that spreads through cat feces or through ingestion of uncooked meat. Infection of *Toxoplasma gondii* leads to fetal injury, with brain and ocular involvement (29). An Iranian survey showed overall seroprevalence of toxoplasmosis is 39.9% (95% CI, 26.1–53.7) among childbearing-age women (30). Identically, IgG of toxoplasmosis is the most sensitive test, because IgM has a high false-positive rate and remains elevated for up to 2 years after infection (31). Our study discovered previous toxoplasmosis infection resulted in a significant decrement in the preterm birth rate [*P* = 0.045, OR = 0.755 (95% CI, 0.571–0.997), adjusted OR = 0.749 (95% CI, 0.566–0.991)], indicating pre-pregnancy toxoplasmosis exposure might be a protective factor in infertile women. The reason for the decreased incidence of preterm birth is uncertain, but might be explained as the presence of antibody prevent individuals from further Toxoplasmosis infection (32). There are no significant differences found in clinical pregnancy, ectopic pregnancy, miscarriage, live birth rate, major malformation, neonatal minor malformation, and perinatal death between the toxoplasmosis IgM (–) IgG(+) group and IgM (–) IgG (–) group, which means patients with previous toxoplasmosis infection don't warrant more attention and further treatments.

Pregnant women without rubella immunity have a high risk of congenital Rubella infection. Fortunately, rubella vaccination has significantly reduced the prevalence of congenital rubella syndrome (CRS) in many countries (33). Serum IgG positivity of rubella implies there is an immune response from the past. No differences in clinical pregnancy, ectopic pregnancy, miscarriage, live birth rate, malformation rate, or perinatal death were noticed between patients with previous rubella infection [IgM (–) IgG(+)] and those without [IgM (–) IgG (–)], indicating rubella IgG may potentially be a protective factor during pregnancy.

HSV infection can be disastrous to newborns. However, neonatal HSV infections are uncommon, occurring in around 1 out of every 3,200 births in the United State considering the high prevalence of HSV infection in the overall population (1). Therefore, routine prenatal screening for HSV infection is not recommended (34). Our study confirmed no differences in clinical pregnancy, ectopic pregnancy, miscarriage, live birth rate, malformation rate, perinatal death between HSV IgM (–) IgG(+) group, and the non-infection group, which is consistent with the advice mentioned above.

This study has limitations. First, despite the large quantity of 18,074 IVF/ICSI-ET cycles included, this study is retrospectively designed, which might have included unmeasured confounding factors and led to potential bias, such as information bias negatively impacting the veracity of the study. Further multi-center prospective studies are needed to provide more convincing evidence. Second, our study merely observed neonatal outcomes; the growth and development of the child were not evaluated. Long-term follow-up of offspring are needed to further explore the effect of maternal previous TORCH infections on offspring health. Third, the results are concluded from clinical data exhibiting a correlation between previous TORCH infection and the pregnancy and neonatal outcomes for couples underwent IVF/ICSI-ET, while we have not yet conducted further basic research to confirm the phenomenon and explore the underlying mechanisms. Last but not least, our study does not investigate the different TORCH pathogen co-infection effects, and further research is needed to distinguish the effect of different co-infected pathogens and to undertake subgroup analysis.

Previous studies have only paid attention to the effects of TORCH infection during pregnancy, and a lot of observations have demonstrated TORCH infection has accounted for several adverse prenatal and neonatal events, including miscarriage, malformation, and neurodevelopmental abnormalities. Our study focuses on a totally different timeframe and a special group of patients to explore whether past infection of TORCH before pregnancy will have an impact on maternal and neonatal outcomes in patients undergoing IVF/ICSI-ET. Our study indicated that previous TORCH infections were not directly associated with adverse pregnancy and neonatal outcomes, providing evidence for clinicians to reduce the screening frequency on this matter. Therefore, further cost-effective analysis might be helpful for clinical strategy of TORCH IgG screening in IVF procedure.

## Data Availability Statement

All datasets generated for this study are included in the article/supplementary material.

## Ethics Statement

The studies involving human participants were reviewed and approved by Hospital Ethics Committee, Women's Hospital, School of Medicine, Zhejiang University. Written informed consent for participation was not required for this study in accordance with the national legislation and the institutional requirements.

## Author Contributions

DZ and GX designed the study and critically revised the manuscript. YL performed data analysis. YW, FW, WZ, LC, ST, YQ, YL, YH, and RZ collected data. YL, YW, FW, and SW drafted the manuscript. All authors reviewed the manuscript.

## Conflict of Interest

The authors declare that the research was conducted in the absence of any commercial or financial relationships that could be construed as a potential conflict of interest.
